# Releasing the neuronal brake: Inhibiting AhR to enhance axon regeneration

**DOI:** 10.1002/ctm2.70733

**Published:** 2026-07-02

**Authors:** Daniel Halperin, Hongyan Zou

**Affiliations:** ^1^ Nash Family Department of Neuroscience, Friedman Brain Institute Icahn School of Medicine at Mount Sinai New York New York USA; ^2^ Department of Neurosurgery Icahn School of Medicine at Mount Sinai New York New York USA

1

Spinal cord injury (SCI) remains a formidable challenge in modern medicine. Despite advances in acute care, complication management and rehabilitation, no effective treatment currently exists to restore axonal connectivity. This therapeutic gap reflects not only a hostile post‐injury microenvironment that impedes axon regrowth, but also an inherent limited ability of injured neurons to re‐enter a repair‐competent state.^1^ Hence, regenerative failure after SCI is broadly attributable to two classes of barriers: the extrinsic barrier encompassing glial scars, myelin‐associated inhibitors, neuroinflammation and extracellular matrix remodelling, and the intrinsic barrier from the diminished neuronal regenerative capacity after SCI.

To dissect the molecular mechanisms governing axon regeneration, the conditioning‐lesion paradigm of dorsal root ganglion (DRG) sensory neuron provides an invaluable experimental platform. DRG neurons have unique features: they extend a peripheral axonal branch into peripheral tissues and a central branch entering the spinal cord. Injury to the peripheral branch triggers a robust regenerative program, whereas central branch injury fails to elicit such a response. However, prior peripheral axotomy places DRG neurons in a “conditioned” state that enables central axon regeneration after SCI.^2^, ^3^ Thus, the regenerative program of adult neurons is not permanently silenced but can be unlocked under the right conditions.

Using the conditioning‐lesion paradigm in DRG neurons, a recent study from our laboratory has identified the aryl hydrocarbon receptor (AhR) as a novel regulator of axon regeneration.^4^ AhR is a basic helix‐loop‐helix transcription factor that functions as a sensor of environmental and physiological cues, binding to a wide variety of compounds, including toxins, xenobiotics, dietary metabolites and endogenous molecules of the tryptophan pathway. Upon ligand binding, AhR translocates to the nucleus and drives transcription of target genes involved in detoxification, stress adaptation, metabolism and cellular homeostasis.^5^, ^6^, ^7^ Although AhR has been characterized in toxicology, immunology, barrier biology and metabolism, its neuronal function, particularly in the context of injury, has not been defined. Through a series of neuronal culture assays and in vivo injury models (peripheral nerve injury and SCI), we demonstrated that AhR inhibition promotes axon regrowth and neural repair.

In mouse DRG neurons, we showed that AhR activation restrains neurite outgrowth, whereas AhR inhibition via gene knockdown, genetic deletion or pharmacological agents enhances axon extension (Figure 1). These effects were also observed in mouse cortical neurons and human induced neurons, indicating a conserved mechanism across neuronal subtypes. In vivo, neuronal AhR deletion enhances axon regeneration and functional recovery following sciatic nerve injury, and in a mouse model of SCI (thoracic contusion), neuronal AhR deletion increases axonal bundles traversing the injury site and improves motor‐sensory outcomes (Figure 1). Critical for clinical translation, pharmacological treatment with StemRegenin 1^8^ (SR‐1, a CNS‐penetrant AhR antagonist) after the onset of SCI can also promote functional recovery.

**FIGURE 1:**
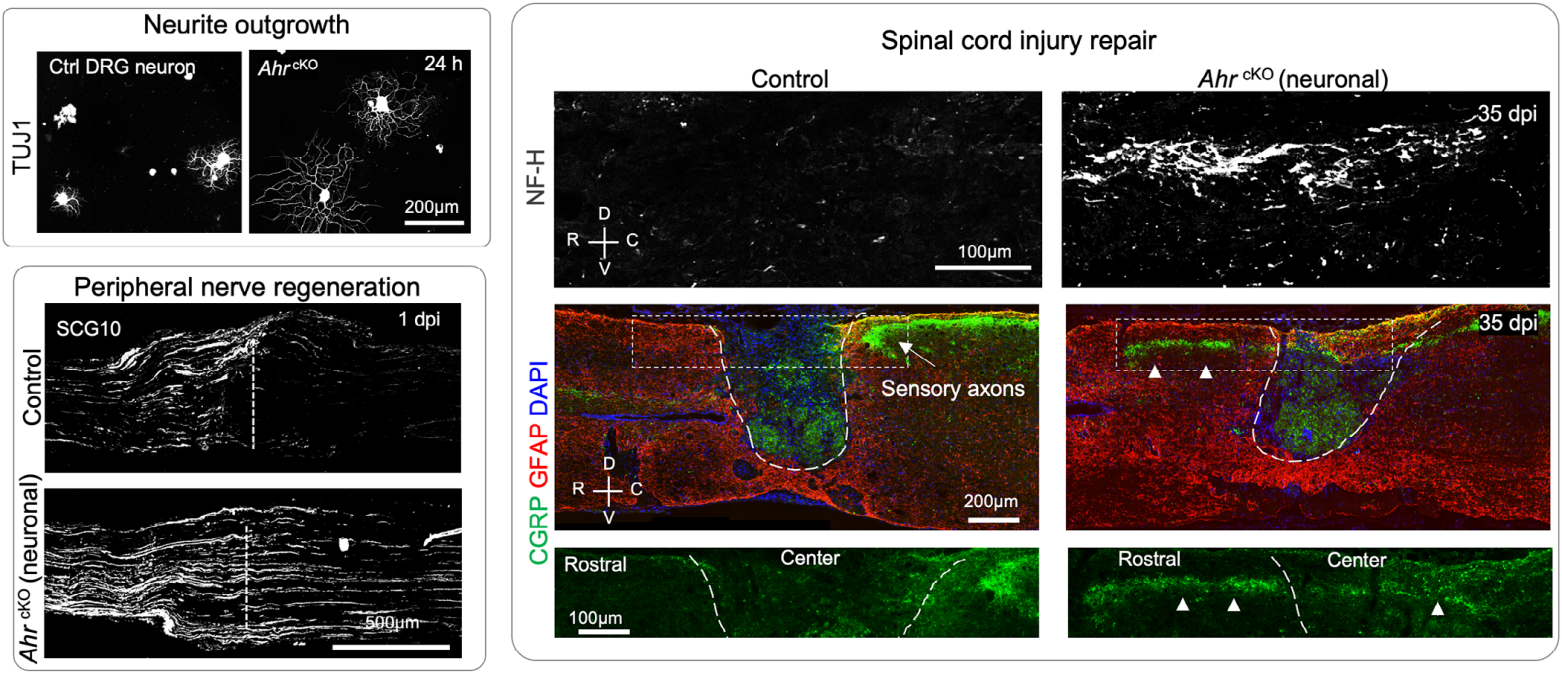
AhR conditional knockout (cKO) in neurons promotes neurite outgrowth and improves axon regeneration in both peripheral nerve and spinal cord injury models. SCG10 labels regenerating sensory axons after peripheral nerve injury, NF‐H labels axon fibres and CGRP labels ascending sensory axons in the dorsal column. Orientation of sagittal spinal cord sections: D, dorsal; V, ventral; R, rostral; C, caudal. Figure adapted from ^4^.

Mechanistically, we demonstrated that axotomy‐activated AhR facilitates stress adaptation by reinforcing proteostasis (protein homeostasis), whereas AhR loss shifts neurons towards de novo protein synthesis, pro‐growth signalling and enhanced mTOR activity—a central regulator of cell growth and translation.^9^ A suitable conceptual framework of these processes is the “stress‐growth switch” model (Figure 2). Following axonal injury, neurons initially activate stress programs to preserve protein quality, metabolic balance and cellular integrity in a hostile microenvironment characterized by inflammation and tissue damage. However, axon regeneration requires a fundamentally different cellular state, one defined by elevated protein synthesis and pro‐growth signalling. Neurons locked in a stress‐containment state may survive the injury but cannot rebuild long‐distance axonal connectivity. This framework may explain the persisting difficulties in developing effective therapeutics: activating a single growth pathway may be insufficient if injured neurons remain locked in a stress‐protective state. By contrast, releasing a neuron‐intrinsic brake such as AhR may unlock multiple repair‐associated programs.

**FIGURE 2:**
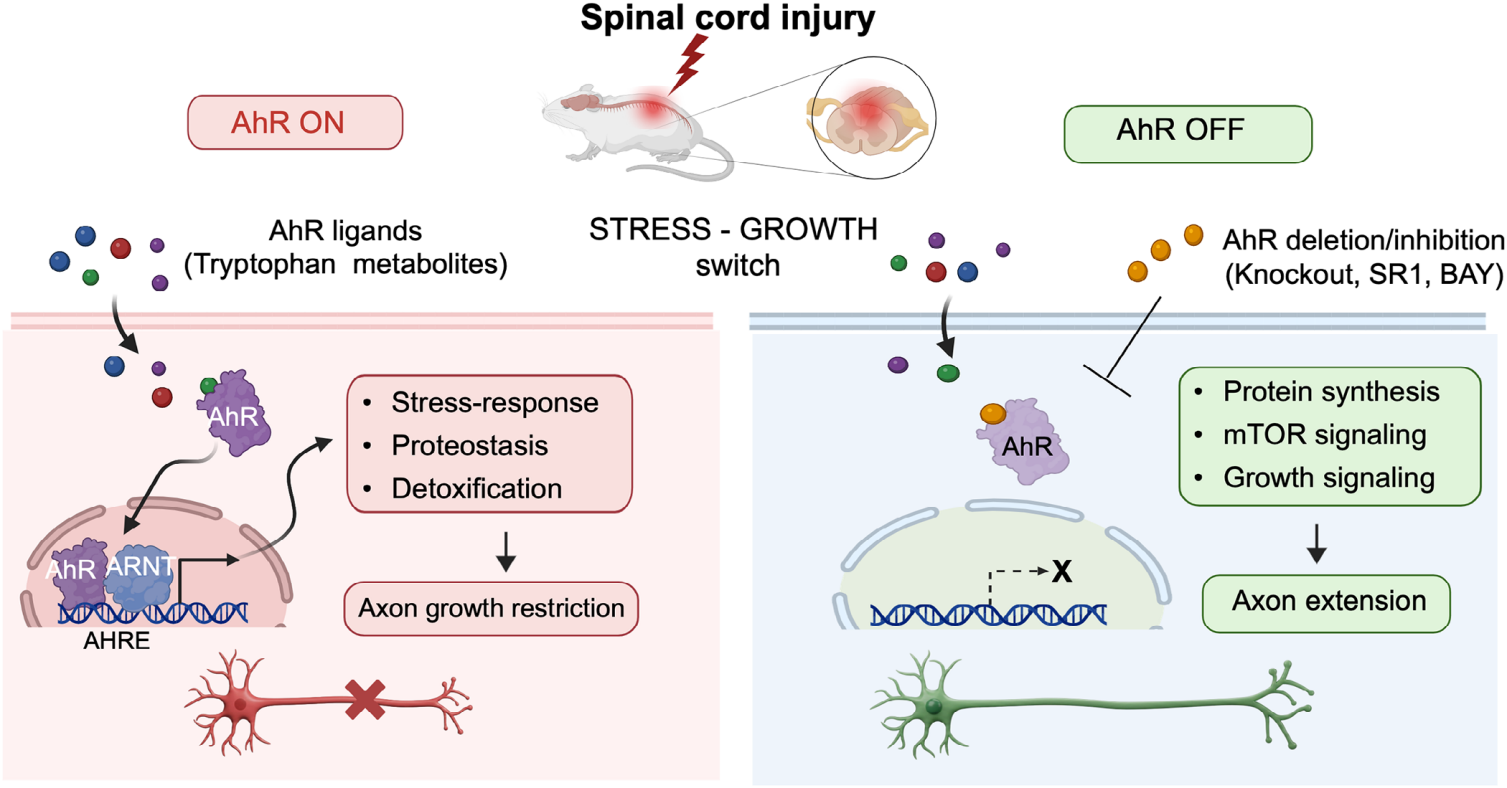
Schematic model of AhR as a stress‐growth switch in neurons after axonal injury. AhR activation (AhR ON) enforces proteostasis, detoxication and stress adaption; AhR inhibition (AhR OFF) releases this neuronal brake to enhance axon regeneration. Figure created using biorender.com.

Because AhR is pharmacologically targetable, our findings position this environment‐sensing pathway as an actionable therapeutic entry point for both peripheral nerve regeneration and SCI repair. Although transcriptional regulators are generally difficult to drug, AhR is ligand‐regulated, making it an attractive pharmacological target. Multiple AhR‐targeting small molecule agents already exist, and AhR antagonism is an emerging strategy for immune modulation and anti‐cancer therapy. For instance, SR‐1 was originally developed to promote expansion of hematopoietic stem cells^8^; in the mouse SCI model, it improves functional recovery, supporting the feasibility of AhR inhibition for CNS injury contexts. Researchers are also engineering selective AhR modulators, which are synthetic molecules optimized via computer‐aided design to selectively inhibit AhR in disease environments while minimizing off‐target effects in healthy tissues.

For clinical translation, AhR inhibition should be conceptualized as a priming strategy for enhancing neuron‐intrinsic regeneration capacity rather than a stand‐alone cure. SCI is a multifactorial condition involving neuronal injury, neuroinflammation, glial scarring, demyelination, vascular disruption, ECM remodelling and disconnection of neural circuits. AhR inhibition may therefore be most effective as one component of a combinatorial repair strategy by increasing the regenerative readiness of injured neurons while complementary interventions modify the lesion environment, provide axon guidance cues, support remyelination or promote activity‐dependent circuit refinement.

The relationship between AhR and other environmental‐sensing pathways may further broaden its therapeutic relevance. The post‐injury spinal cord is metabolically and immunologically complex, exposing neurons to hypoxia, immune mediators, myelin debris and endogenous metabolites. Notably, AhR shares its dimerization partner ARNT with hypoxia response master regulator HIF‐1α, and prior work has linked hypoxia signalling to axon regeneration.^10^ These connections suggest that injured neurons integrate multiple environmental cues before committing to a regenerative state, and AhR may serve as a convergence point for this decision.

Several challenges must be addressed before clinical translation. AhR signalling is context‐dependent, and systemic inhibition of AhR may perturb immunity, metabolism and barrier functions. Determining optimal treatment windows, dosing, blood‐spinal cord barrier penetration and durability of effect will also be needed. Nevertheless, the conceptual advance by this work demonstrates that axon regeneration can be promoted not only by extrinsic growth factors but also by removing intrinsic restraints that lock injured neurons in a protective, non‐regenerative state. This insight provides a starting point for future SCI repair strategies based on AhR blockade to initiate a stress‐growth switch in injured neurons, followed by supplying supportive environmental cues, guidance molecules and activity‐dependent signals to reconnect and fine‐tune the disrupted neuronal circuits.

## AUTHOR CONTRIBUTIONS

Daniel Halperin and Hongyan Zou prepared the manuscript.

## CONFLICT OF INTEREST STATEMENT

The authors declare no conflict of interest.
